# The Effects of *Momordica charantia* on Type 2 Diabetes Mellitus and Alzheimer’s Disease

**DOI:** 10.3390/ijms24054643

**Published:** 2023-02-28

**Authors:** Erika Richter, Thangiah Geetha, Donna Burnett, Tom L. Broderick, Jeganathan Ramesh Babu

**Affiliations:** 1 Department of Nutritional Sciences, Auburn University, Auburn, AL 36849, USA; 2 Boshell Metabolic Diseases and Diabetes Program, Auburn University, Auburn, AL 36849, USA; 3 Department of Physiology, Laboratory of Diabetes and Exercise Metabolism, College of Graduate Studies, Midwestern University, Glendale, AZ 85308, USA

**Keywords:** bitter melon (*M. charantia*), type 2 diabetes mellitus, Alzheimer’s disease, glucose-lowering effects, hypoglycemic, medicinal plants, natural, neuroprotective, prevalence, therapeutic, bioactive

## Abstract

T2DM is a complex metabolic disorder characterized by hyperglycemia and glucose intolerance. It is recognized as one of the most common metabolic disorders and its prevalence continues to raise major concerns in healthcare globally. Alzheimer’s disease (AD) is a gradual neurodegenerative brain disorder characterized by the chronic loss of cognitive and behavioral function. Recent research suggests a link between the two diseases. Considering the shared characteristics of both diseases, common therapeutic and preventive agents are effective. Certain bioactive compounds such as polyphenols, vitamins, and minerals found in vegetables and fruits can have antioxidant and anti-inflammatory effects that allow for preventative or potential treatment options for T2DM and AD. Recently, it has been estimated that up to one-third of patients with diabetes use some form of complementary and alternative medicine. Increasing evidence from cell or animal models suggests that bioactive compounds may have a direct effect on reducing hyperglycemia, amplifying insulin secretion, and blocking the formation of amyloid plaques. One plant that has received substantial recognition for its numerous bioactive properties is *Momordica charantia (M. charantia)*, otherwise known as bitter melon, bitter gourd, karela, and balsam pear. *M. charantia* is utilized for its glucose-lowering effects and is often used as a treatment for diabetes and related metabolic conditions amongst the indigenous populations of Asia, South America, India, and East Africa. Several pre-clinical studies have documented the beneficial effects of *M. charantia* through various postulated mechanisms. Throughout this review, the underlying molecular mechanisms of the bioactive components of *M. charantia* will be highlighted. More studies will be necessary to establish the clinical efficacy of the bioactive compounds within *M. charantia* to effectively determine its pertinence in the treatment of metabolic disorders and neurodegenerative diseases, such as T2DM and AD.

## 1. Introduction

Diabetes mellitus is considered one of the five leading causes of death in the world affecting an estimated 537 million adults in the year 2021 [1,2]. By 2045, this number is expected to rise to 783 million worldwide [2,3,4]. There are two major types of DM, the first being type 1 diabetes (T1DM), characterized by hyperglycemia due to the autoimmune destruction of pancreas beta cells, resulting in the overall decreased production of insulin [3,4,5]. The more common version of this metabolic disorder is type 2 diabetes mellitus (T2DM), described by increased insulin release to compensate for insulin resistance and progressive decline in islet secretory function within the pancreas, thus causing overall insulin deficiency [3,4].

Several complications can result from diabetes, including nephropathy, retinopathy, neuropathy, and atherosclerosis [3,4]. About 60–70% of diabetes patients will eventually develop peripheral neuropathy if proper care and treatment do not take place [6]. If left untreated, these complications may become life-threatening and lead to heart disease, kidney disease, blindness, and in some cases amputations.

Recent epidemiological research proposes the likely association between the development of Alzheimer’s disease (AD) and diabetic neuropathy arising from T2DM [7]. In fact, AD is now being referred to as “type 3 diabetes”, as it is considered a form of diabetes that selectively involves the brain and has molecular and biochemical features that intersect with both T1DM and T2DM [8]. AD is characterized as the gradual deterioration of the brain, gravely impairing neurological function leading to symptoms of early onset dementia and impacting one’s ability to carry out simple everyday tasks [9]. An estimated 5.8 million Americans were living with AD in 2020 [5]. Due to rampantly increasing rates of AD, coupled with high economic and social stress, it is imperative to secure successful, yet practical treatment options to effectively delay the symptoms and development of both T2DM and AD [3]. Antidiabetic drugs have various adverse effects; however, there has been no success in finding a treatment that effectively prevents or reverses AD progression [7,9]. As AD has no cure, it is essential to determine whether any antidiabetic drugs or therapies hold any significant improvement toward the progression or prevention of the disease, which is where nutrition plays a vital role [10,11].

Considering the underlying biochemical associations between AD and T2DM, it is important to thoroughly investigate preventative measures from a nutritional standpoint, as there may be a common therapeutic target for AD and T2DM within the bioactive components of certain medicinal foods [12,13]. These components found within nature can be found in several plants, many of which are consumed in the everyday diet across the globe [12,13]. Through the consumption of a variety of different fruits, vegetables, plants, and animal proteins, an array of vitamins, minerals, and phytochemicals are provided for the body. 

A recent study has unveiled that an estimated 30% of patients with DM use alternative medicine as a way of controlling their metabolic state [3,14]. Therefore, identifying the bioactive components within these plants that aid in the control or delay of diseases such as AD and T2DM is the next step in finding affordable and preventative medicine. With food functioning as medicine, the prevention of these diseases is attainable from both an economic and scientific standpoint. Identifying the biochemical elements within these nutriments which hold such curative power is the first step in establishing nutrition as a modern, effective medicine. This tactic is otherwise known as complementary and/or alternative medicine and utilizes herbs and other dietary subsistence from various plants and animals secondary to traditional medical treatments [3]. Through further research on the bioactive components within food, modern-day medicine can expand its treatment approach towards metabolic disease states such as T2DM and AD. Further, the natural bioactive compounds within foods can be utilized as an accessible, promising dietary treatment option for DM and AD due to the efficacy, few side effects, and availability of the nutritional components [15]. 

*M. charantia*, otherwise known as bitter melon, karela, balsam pear, or bitter gourd, is a popular plant commonly used to treat diabetes-related conditions amongst the indigenous populations of Asia, South America, India, the Caribbean, and East Africa [3,16,17]. This odd gourd is known for its distinguishing bitter taste, which becomes more distinct through maturation [3]. Through several biochemical and animal model experiments, an ample amount of data and hypotheses have been procured regarding the antidiabetic effects of *M. charantia*, although very few clinical human studies with adequate design quality have been published [3]. However, the effects of *M. charantia* on the treatment of AD have yet to be adequately explored. As the connection between DM and AD grows seemingly more intertwined, nutritional preventative care needs to be at the forefront of research. Further clinical research with a heightened focus on the nutritional aspect of medicinal foods such as bitter melon cannot only help expand preventative treatment options for the population but also help alleviate the economic burden these metabolic diseases create today. 

Gaining specific knowledge regarding the bioactive components found in *M. charantia* would provide the necessary insight for the proper strategic consumption and sustainable use needed to enhance the treatment of both T2DM and AD [3]. By further understanding the capabilities of the nutritive elements within various plant species, their potential role in overall health strengthens allowing nutrition to work in congruence or replace common T2DM and AD pharmaceuticals. Thus, utilizing the antidiabetic and hypolipidemic effects of *M. charantia* in congruence with modern medicine may be an effective option to best treat DM as well as delay further complications of the disease, including the progression of AD [3]. Furthermore, investigating the various antioxidant, anti-inflammatory, and antiapoptotic properties of *M. charantia* may help shed light on the importance of incorporating nutrition as part of preventative care as a whole [1,15,18]. 

Through the assessment of the various beneficial effects of bioactive compounds found in *M. charantia* and other medicinal plants, their impact on overall health can be better understood. By doing so, medical nutrition therapy increases the number of effective methods readily available for the treatment of metabolic disease states making large strides in advancing medicine for the benefit of the overall health and well-being of the population [1]. Throughout this paper, the overall medicinal impact of *M. charantia* will be further understood through a discussion of the pathophysiology of T2DM and AD, and how they relate to one another, as well as the underlying mechanisms of the bioactive components that make up *M. charantia.* Through this detailed review, the potential antidiabetic, and hypoglycemic effects of *M. charantia* and its medicinal potency in effectively treating T2DM and AD will be thoroughly discussed. 

## 2. Pathophysiology of T2DM and AD

To fully understand the beneficial effects of the bioactive compounds of bitter melon on T2DM and AD, we must first discuss the pathophysiology of both diseases [3]. 

### 2.1. Type 2 Diabetes Mellitus

Insulin resistance coupled with an overproduction of hepatic glucose, along with dysfunctional β-cell activity, thus leading to β-cell deficiency characterizes the onset of T2DM [19,20]. Peripheral insulin resistance is attributed to the subsequent failure of cells to efficiently react to changing insulin levels within the various target tissues within the body [21]. In the body’s normal state, insulin will suppress the production of glucose from the liver during both fasting states and after a meal is consumed [22]. However, in the case of insulin resistance, glucose levels tend to increase after a meal rather than becoming corrected back down to a normal postprandial state causing hyperglycemic episodes indicating T2DM [22]. Another major factor contributing to elevated hepatic glucose production is heightened lipolysis within fat cells [23]. Initially, insulin resistance will trigger compensatory ß-cell hypertrophy and a rise in insulin production; however, continued subjection to hyperglycemic-induced oxidative stress, inflammatory markers, and immune-suppressing cell behaviors may add to the subsequent cell death and diminished cell expansion leading to overall β-cell destruction [24,25]. This continuous decline of β-cell performance eventually reduces insulin secretion, thus disrupting glucose homeostasis [25]. There are several pharmacotherapy treatment options available to control T2DM; however, recent studies also suggest that healthy lifestyle choices may prove to be more effective at prevention and treatment in the long run [26].

### 2.2. Alzheimer’s Disease

AD is a progressive neurological disease characterized by brain atrophy and subsequent dementia affecting one’s overall quality of life and ability to function independently [8]. Early signs of the disease include short-term memory loss and, as the disease progresses, serious disruptions in the ability to carry out simple everyday tasks ensue [8]. AD can be distinguished from other metabolic disease states by the accumulation of extracellular misfolded amyloid plaques (Aβ peptide) in senile plaques, intracellular neurofibrillary tangles (NFTs), inflammation of neurotransmitters, and the deterioration of specific areas within the cerebrum [8,13,27]. Aβ peptides are made up of 38–43 amino acid residues originating from a chemical change in the amyloid precursor protein (APP) [19,28]. In normal states, APP utilizes an α-secretase to create Aβ products that tend to not produce amyloid deposits [19,28,29,30]. However, in a brain negatively affected by AD, BACE-1 (a β-secretase), and γ-secretase assemble Aβ from APP through several overriding enzymatic steps [19,28,29,30]. The failure to remove excess Aβ, therefore, gives rise to the aggregation of Aβ and the ensuing damage to the nervous system as a whole [8,30]. Following this event, the Aβ’s will then spontaneously manifest into various configurations, including undesirable monomers, oligomers, fibrils, and plaques [19,31]. Another common indicator of an AD diagnosis is the detection of NFT within the brain [29]. Although medications may temporarily improve or slow the progression of symptoms, there has yet to be a successful treatment that fully cures this deadly disease [8]. Therefore, it is imperative to look beyond traditional medications to make meaningful strides in medicine.

## 3. Common Link between T2DM and AD

Although T2DM and AD seem like two completely different disease states with entirely different sets of symptoms and complications, this is not the case. The biochemical, molecular, and cellular abnormalities that precede or accompany AD neurodegeneration, including heightened activation of predeath genes and signaling pathways, impaired energy metabolism, mitochondrial dysfunction, chronic oxidative stress, and DNA damage, are all recognizable detriments with the exact etiologies remaining unknown [8,32,33,34,35,36,37,38,39]. Recent evidence suggests a role of impaired cerebral glucose utilization and energy metabolism, which represent very early abnormalities that coincide with the initial stages of cognitive impairment in AD [8,40,41,42]. This finding has led researchers to believe that impaired insulin signaling has an important role in the pathogenesis of AD, thus its potential representation as “type 3 diabetes” [8,33]. Therefore, both T2DM and AD are intertwined through several characteristics, including chronic inflammation, oxidative stress, impaired insulin signaling, insulin resistance, glucose intolerance, and cognitive impairment [12]. By further understanding what links the two of these disease states, we will be able to better understand the underlying mechanisms of the bioactive components of plants such as *M. charantia* and their impact on both T2DM and AD.

### 3.1. Insulin Resistance 

**Figure 1:**
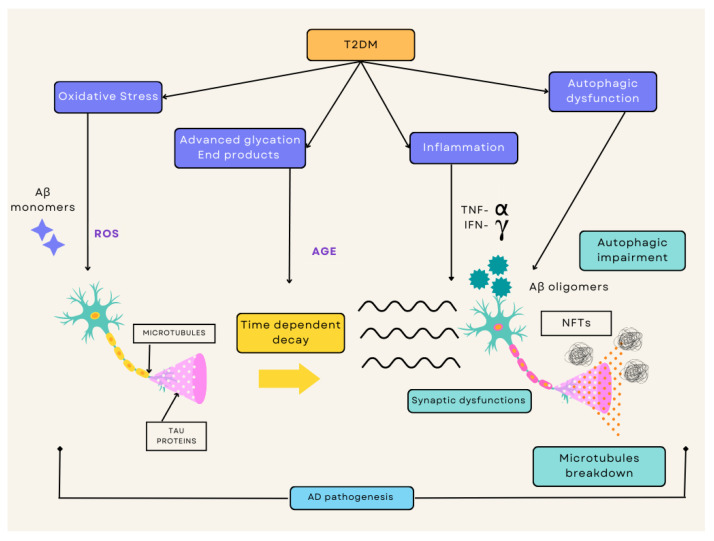
Overview of the diverse mechanisms by which T2DM and AD crossover with one another. This consists of a cascade of pathogenic processes including oxidative stress, advanced glycation products, inflammation, and autophagic dysfunction. The ROS cultivated by these pathways increases apoptosis occurrence, whereas the IR simultaneously disturbs the signaling pathways. This causes an increased formation of Aß oligomers and aggregated hyperphosphorylated tau. This process leads to overall degradation with synaptic dysfunction, autophagic impairment, and microtubule breakdown, thus resulting in neuronal death. T2DM, type 2 diabetes mellitus; AD, Alzheimer’s disease; IR, insulin resistance; ROS, reactive oxygen species.

Recent emerging evidence has revealed that the markers of insulin resistance and insufficiency, prevalent in T2DM, are also contributors to the pathology of AD [19,43]. Consequently, AD is denoted as a form of DM targeting the nervous system [13,19]. Insulin receptors (IR) are expressed in both the peripheral and central nervous systems (CNS), which include a region of the brain called the hippocampus [19,44,45]. The hippocampus is largely responsible for learning and memory function and is most often the earliest affected area in AD neurological degradation [19,44,45]. This occurs due to the attachment of insulin to IR leading to the phosphorylation of tyrosine and the subsequent stimulation of insulin receptor substrate (IRS), thus enhancing the activity of both Akt and phosphatidylinositol-3 kinase (PI3 kinase) [19,28]. Akt will then mediate the phosphorylation or deactivation of glycogen synthase kinase 3β (GSK3β) [19,28]. This results in lowered insulin signaling causing an increased GSK3β activity [19,28,46]. This phenomenon causes the hyperphosphorylation of tau proteins, the emergence of NFTs, and heightened assembly of Aβ within the cerebrum, as illustrated in Figure 1 [19,28,46]. In a brain affected by AD, Aβ oligomers accelerate atypical activation of tumor necrosis factor-α (TNF-α)/c-Jun N-terminal kinase pathway (JNK), thus bringing about the inhibition of IRS1 and the overall disturbance in insulin regulation [12,19,47]. Furthermore, the insulin-degrading enzyme (IDE) regulates the breakdown of Aβ and APP, creating competition between insulin and Aβ [19,48], and therefore reducing Aβ destruction altogether regarding insulin resistance [18,19].

T2DM accompanied by IR and hyperglycemia increases the risk of potential metabolic issues within the brain and other tissues that initiates a cascade of pathogenic processes such as oxidative stress, inflammatory responses, advanced glycation products, and autophagic dysfunction. The ROS cultivated by these pathways heightens the process of neuronal death. Simultaneously, the IR disturbs the signaling pathways and causes an increased formation of Aß oligomers and aggregates of hyperphosphorylated tau. The overall effect of all these factors allows neurons to face serious degradation, eventually resulting in the loss of synapses and neuronal death (Figure 1).

### 3.2. Chronic Inflammation

Chronic inflammation is another pertinent link between T2DM and AD [19]. Elevated erythrogenic cytokines, including interleukin-6 (IL-6), interleukin-1β (IL-1β), and TNF-α are recognized markers of T2DM [19,49]. The emergence of these inflammatory proteins is correlated with β-cell destruction and death, as well as diminished insulin production, all of which negatively affect the pancreas and/or brain [19,50,51]. Certain proinflammatory cytokines also have been found to cross the blood–brain barrier (BBB), causing harmful effects on the CNS, thus leading to the postulated induction and progression of AD [19,52]. In a recent study, higher amounts of IL-1 within the cerebrum led to limited acetylcholine (Ach) delivery, decreased nerve growth factor (NGF) expression, and problems with memory recollection [19,52,53]. Another critical aspect of chronic inflammation involves advanced glycation end products (AGE), produced through spontaneous non-enzymatic reactions of free reducing sugars with free amino groups of various proteins and lipids [19,54]. In cases of chronic hyperglycemia seen in T2DM, AGEs are further generated by interacting with RAGE receptors, prompting the activation of various intracellular erythrogenic pathways such as the nuclear factor-kappa B (NF-κB) and proinflammatory mediators such as IL-6, TNF-α, and C-reactive protein (CRP) [19,54,55]. These AGEs noticeable in T2DM may also be involved in AD pathology due to Aβ attachment to RAGE [3]. This occurrence promotes Aβ crossing through the BBB, causing Aβ inflation linked to AD development [19,56,57]. Additionally, this interaction relates to elevated levels of oxidative stress, magnified inflammatory response, and severe damage to neuronal cells [19,58].

### 3.3. Oxidative Stress

Oxidative stress ensues from a chemical disparity between the making of reactive oxygen species (ROS) and reactive nitrogen species (RNS), as well as antioxidant defense, thus attributing to the rise of T2DM and AD development [19,59]. The disruption of crucial polyunsaturated fatty acids (PUFAs), proteins, and DNA within cell membranes can be attributed to some of these ROS including nitric oxide synthase (iNOS), cyclooxygenase-2 (COX-2), and hydroxyl radicals [19,60]. One of the main factors contributing to the ongoing development of T2DM and its complications includes the overproduction of these ROS/RNS entities [4,19]. In cases of T2DM, elevated glucose levels may promote glucose autoxidation, mitochondrial defects, and heightened levels of ROS [3,53]. Excess ROS promotes lipid peroxidation causing subsequent β-cell failure and weakened biochemical pathways, such as NF-κB, JNK/stress-activated protein kinase (SARK), and p38-mitogen-activated protein kinase (p38-MAPK), thus contributing to IR and metabolic complications [19,61,62]. These factors hold a significant role in the prognosis of AD as well [19,61,62]. Since neurons heavily rely on mitochondria for ATP production and maintenance of Ca^2+^ homeostasis, any oxidative stress on these mitochondrial pathways may result in neuronal injury and death due to bioenergetic depletion [19,63]. Moreover, mitochondrial damage can also enhance ROS production, which then amplifies NFT development, tau hyperphosphorylation, and Aβ aggregation, which promote the overall generation of oxidative stress [19,61]. Together, these factors accelerate the progression of AD, thus making it imperative to find an effective treatment that prevents oxidative stress [19]. 

By further investigating the common biochemical abnormalities that T2DM and AD share, a common therapeutic and preventive agent that may be effective in treating both diseases can be found [3]. The medicinal properties of *M. charantia* may be a potential solution to preventing and slowing down the progression of insulin resistance, chronic inflammation, and oxidative stress that occurs in these metabolic disorders. 

## 4. The Potential of Plant-Based Medicine

For thousands of years, *M. charantia* has been used as an herbal remedy throughout many countries and regions, as its components provide impactful beneficial effects [64]. The plant itself, especially its fruit and seeds, contains significant pharmacological effects that have been utilized in the treatment of DM as well as AD as recent evidence suggests its plausible role in its pathology [64,65]. The phytoconstituents within bitter melon activate antihyperglycemic, antioxidant, antidiabetic, hepatoprotective, antibacterial, and anti-inflammatory activities in tissues and cellular pathways [64,65,66,67,68,69,70].

### 4.1. Impact of Plant-Based Medicine

Plant-based medicine is a cost-effective, safe, and practicable option to treat T2DM and AD [3]. In fact, in many developing parts of the world, this may be the only therapeutic option for medical treatment [3]. There are several studies reviewing the effectiveness of antidiabetic herbal plants in comparison to modern-day forms of medicine [71,72,73,74,75,76,77]. The ancient Indian medical system, otherwise known as Ayurveda, utilizes several plants and herbs for the treatment of disease states such as T2DM and AD, as this system is based on a natural and holistic approach to both physical and mental overall health [3]. Although this method is not considered modern medicine, it is a system that produces few side effects at a low cost [3].

### 4.2. Utilization of Bitter Melon as Plant-Based Medicine

**Figure 2:**
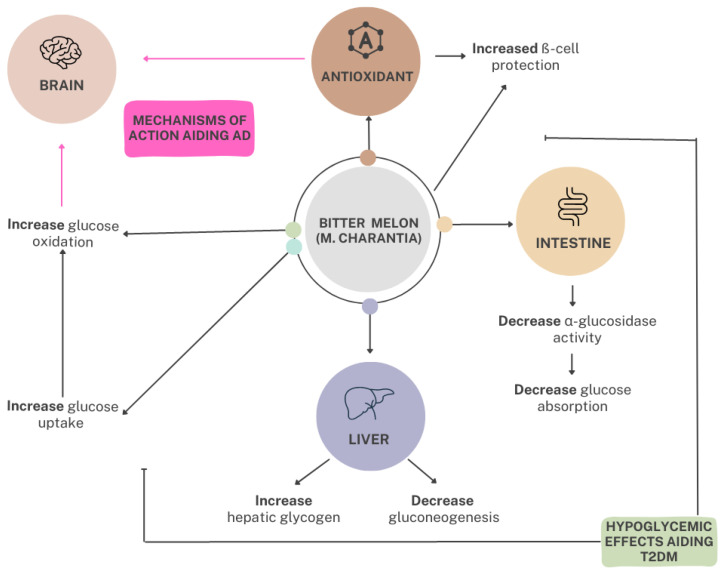
The mechanisms of action of bitter melon in the reduction of blood glucose in relation to T2DM and AD. M. charantia maintains hypoglycemic effects through antioxidant effects promoting ß-cell protection and the decrease in glucose absorption from the intestines and glucose production from the liver by increasing hepatic glycogen and decreasing gluconeogenesis. Thus, from the consumption of bitter melon, glucose uptake and oxidation within the brain are impacted, directly affecting the brain’s energy source. The overall effect of these mechanisms influences the prevention and treatment of T2DM and AD. T2DM, type 2 diabetes mellitus; AD, Alzheimer’s disease.

Current diabetes medications utilize the insulin and oral hypoglycemic effects within *M. charantia* to control T2DM and other metabolic conditions. Metformin, one of the most popular diabetic medications, is one of them. This medication lowers blood sugar levels by improving insulin sensitivity. *M. charantia* maintains the same hypoglycemic effects as metformin; it promotes insulin secretion, improving glucose uptake by adipose or muscle tissues, and inhibiting glucose absorption from the intestines and glucose production from the liver [78], whereas consuming *M. charantia* or its extracts can present the same effects with far fewer side effects [3]. Current research exemplifies the potential use of *M. charantia* as a more recognizable tool for T2DM and AD prevention and treatment, as it is the most accepted hypoglycemic plant [3]. M. *charantia* maintains hypoglycemic effects through antioxidant effects promoting ß-cell protection, the decrease in glucose absorption from the intestines and glucose production from the liver, and improved glucose uptake by adipose or muscle tissues, thus affecting the brain as well due to glucose being its main energy source as illustrated in Figure 2 [79]. This paper will focus on *M. charantia* and its bioactive components in relation to its ability to control, treat, and possibly prevent both T2DM and AD, a seemingly interconnected disease state due to impaired insulin signaling. Although bitter melon’s potential as a replacement therapy for traditional medicine still needs further clinical research, the result of current research looks promising [3].

## 5. The Profile of Bitter Melon

With a high vitamin and mineral content, *M. charantia* is one of the most promising medicinal plants available for not only the treatment of T2DM and AD but for overall human health and longevity [3,29]. Distinguishable by its unusual taste, bitter melon may be further explained by its various bioactive effects that general fruits and vegetables do not provide [64]. Though there are several herbal remedies that claim to effectively treat T2DM and AD, *M. charantia* is one that has received a considerable amount of attention [3]. Traditionally, bitter melon has been known for its effectual antidiabetic, anticancer, anti-inflammation, antivirus, and cholesterol-lowering medicinal properties [3,64]. Antioxidant and antimutagen properties have also been identified amongst *M. charantia’s* of bioactive and phenolic compounds [3,15,44]. 

### 5.1. Bitter Melon Description

*M. charantia*, otherwise known as bitter melon or bitter gourd, is a member of the Cucurbitaceae family distinctly known for its intensely bitter taste, as shown in Table 1 [3]. This flowering vine is widely cultivated in tropical and subtropical regions of the world such as Asia, India, East Africa, and South America [3,64]. This climbing perennial can grow up to 5 m tall and produces oblong or spindle-shaped fruits with knobby, protruding bumps along the surface [3,28,68,69]. Resembling a small cucumber, bitter melon’s looks deceive. Its exterior starts out a deep emerald green and as it ripens turns bright orange, while the inside of the fruit changes to bright red during maturation [64]. 

The fruit itself can be incorporated into the diet in every stage of maturation and can be found in several popular dishes [64]. In Asian culture, bitter melon is incorporated into recipes with knowledge of its medicinal capability, whereas in Western culture the notion regarding bitter melon is not as recognized. As the population becomes more aware of the benefits of a well-rounded diet, trends in overall health will reap the benefits. Thus, through a nutritious diet with foods such as *M. charantia*, certain metabolic disease states such as T2DM and AD can be better controlled, treated, and prevented. 

### 5.2. Nutrient Profile

**Figure 3:**
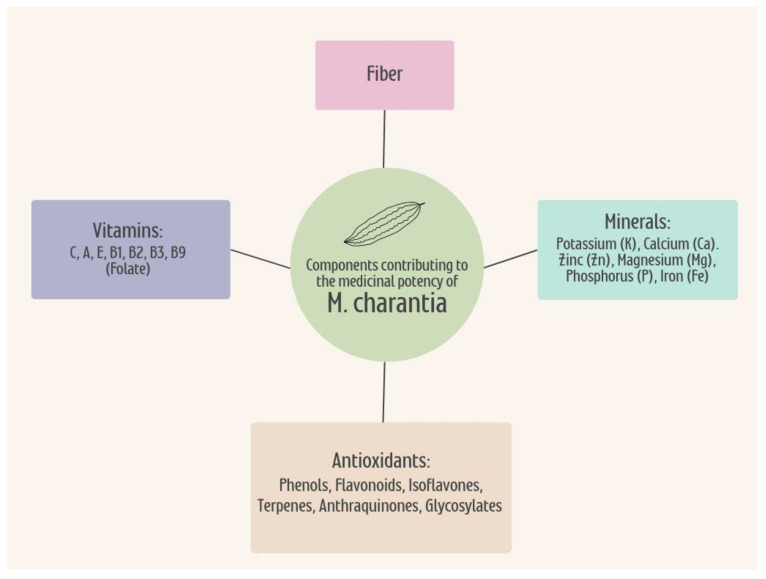
The combination of the nutritional properties within M. Charantia contributes to the overall therapeutic potential for the treatment and prevention of T2DM and AD. The vitamins, minerals, antioxidants, and fiber hold medicinal potency from the plant when consumed in the diet, as well as providing nutrients benefitting overall health. T2DM, type 2 diabetes mellitus; AD, Alzheimer’s disease.

*M. charantia* is a potent nutrient-dense plant composed of an elaborate range of beneficial compounds and elements [3]. The powerful assembly of bioactive compounds, vitamins, minerals, and antioxidants within this gourd all give rise to its remarkable ability in treating a wide range of illnesses [3]. Bitter melon contains large amounts of vitamins C, A, E, B1, B2, and B3, as well as vitamin B9 (folate), making this vegetable a healthful addition to any diet [3]. Regarding caloric content, the values for the leaves, fruit, and seeds are approximately 213, 242, and 177 Kcals per 100 g [80]. Bitter melon is also rich in many minerals including potassium (K), calcium (Ca), zinc (Zn), magnesium (Mg), phosphorus (P), and iron (Fe), and is an excellent source of dietary fiber [3]. A diet rich in vitamins, minerals, and fiber can help promote the overall health and well-being of the general population, especially those at risk of developing metabolic disorders, such as T2DM and AD. The medicinal effects of *M. charantia* can be explained by its high antioxidant properties due in part to components such as phenols, flavonoids, isoflavones, terpenes, anthraquinones, and glycosylates, all of which contribute to its formidable bitter taste [3,81]. All these properties combined are what give bitter melon such therapeutic potential for treating and preventing T2DM and AD, as well as providing adequate nutrition, vitamins, and minerals to those who incorporate this unusual vegetable into their diet [Figure 3]. 

### 5.3. Phytochemistry

*M. charantia* is made up of several carbohydrates, proteins, and lipids that all possess different bioactive components that prove to be beneficial for overall health and specific ailments for certain metabolic diseases [64]. Within these classifications lie various key bioactive components such as triterpenoids, saponins, polypeptides, flavonoids, alkaloids, and sterols which are the compounds ultimately responsible for these medicinal effects that bitter melon is so well known for shown in Table 2 [3,64,82,83]. 

*M. charantia* also consists of glycosides, saponins, reducing sugars, resins, phenolic constituents, fixed oil, and free acids [3,156]. *M. charantia* consists of the following chemical constituents: alkaloids, charantin, charine, cryptoxanthin, cucurbitins, cucurbitacins, cucurbitanes, cycloartenols, diosgenin, elaeostearic acids, erythrodiol, galacturonic acids, gentisic acid, goyaglycosides, goyasaponins, guanylate cyclase inhibitors, gypsogenin, hydroxytryptamines, karounidiols, lanosterol, lauric acid, linoleic acid, linolenic acid, momorcharasides, momorcharins, momordenol, momordicilin, momordicin, momordicinin, momordicosides, momordin, momordolo, multiflorenol, myristic acid, nerolidol, oleanolic acid, oleic acid, oxalic acid, pentadecans, peptides, petroselinic acid, polypeptides, proteins, ribosome-inactivating proteins, rosmarinic acid, rubixanthin, spinasterol, steroidal glycosides, stigmasta-diols, stigmasterol, taraxerol, trehalose, trypsin inhibitors, uracil, vacine, v-insulin, verbascoside, vicine, zeatin, zeatin riboside, zeaxanthin, zeinoxanthin amino acids-aspartic acid, serine, glutamic acid, thscinne, alanine, g-amino butyric acid and pipecolic acid, ascorbigen, b-sitosterol-d-glucoside, citrulline, elasterol, flavochrome, lutein, lycopene, and pipecolic acid. The pulp within bitter melon contains soluble pectin but no free pectic acid [3]. Recent evidence indicates that the leaves of bitter melon are significant nutritional sources of Ca, Mg, K, P, and Fe. It was also demonstrated that both the edible portion of *M. charantia*, along with the leaves are excellent sources of B vitamins, which are significant in overall health and longevity [3,79].

Further understanding the phytochemical composition of the bioactive elements within *M. charantia* will allow the specific role and impact of each constituent to be easily identified. As research continues to isolate the components of *M. charantia,* the more likely this “superfood” is to be utilized as a recognized method of medical nutrition therapy for patients suffering from various metabolic disorders. By doing so, bitter melon can be used as a cost-effective method in the treatment, prevention, and control of both T2DM and AD.

## 6. The Bioactive Compounds of Bitter Melon

Bioactive compounds are the components within foods that can regulate the metabolic processes in both humans and/or animals and improve overall health [19,62]. These bioactive components are found largely in vegetables, fruits, and whole grains, which can be consumed daily with a healthy diet that consists of a variety of different foods [20,64]. These bioactive components have the potential to regenerate pancreatic β cells, enhance insulin release, and reverse insulin resistance, all of which are desirable in the control and prevention of several metabolic disease states [77].

The beneficial effects of various bioactive compounds within *M. charantia* have been found to diminish inflammation, target free radicals, and regulate cell signaling pathways in both cell and animal studies [19,63,64]. Due to their rich availability, safety, and low amount of side effects, the use of bioactive compounds has been reported to lessen the occurrence or delay the progression of several diseases, including T2DM and AD [15,18,19]. Some examples of bioactive compounds include polyphenols, carotenoids, phytosterols, polysaccharides, and vitamins [19].

The role of major compounds that have been isolated from bitter melon and identified as hypoglycemic agents include polysaccharides; proteins and peptides such as polypeptide-p, and peroxidase; saponins and terpenoids such as charantin; and flavonoids and phenolic compounds such as quercetin, rutin, kaempferol, and isorhamnetin —[3,64,84,157], Table 2. These bioactive constituents within bitter melon provide it with medicinal properties, thus making it essential to fully assess their mechanisms of action in detail to successfully utilize the plant and its extracts in actual, medicinal practice.

For AD in particular, recent research has revealed the neuroprotective impact of bioactive compounds within natural products, such as bitter melon, through its influence on the peripheral metabolism affecting the cognitive function of the brain, whereas traditional drugs must target crossing the BBB to be effective [158]. Additionally, when a drug is ineffective, it is likely due to its inability to pass the BBB [158]. Thus, further research on the bioavailability of natural products, such as *M. charantia,* is essential in determining its impact on the CNS and its therapeutic potential on AD. The bioactive compounds within natural products have an impact on brain function but work differently than typical medications as they do not necessarily cross the BBB [158]. This next section will discuss the effects of each of these bioactive compounds within the inner workings of metabolism and the CNS, along with their mechanisms of action.

### 6.1. Polysaccharides

Polysaccharides are carbohydrates consisting of small sugar molecules that formulate the starch, cellulose, or glycogen stores of a plant, and are one of the more important bioactive components of *M. charantia* [64]. Carbohydrates are not only utilized as an energy source for the brain, but also heavily influence biological interactions affecting cell growth, signaling, and differentiation. From this information, research has revealed the potential of the natural carbohydrates from the fruit of *M. charantia* to possess various antioxidant, antidiabetic, immune, neuroprotective, antitumor, and antimicrobial bioactivities [141,142,143,144,145,146,147]. Polysaccharides within bitter melon are classified as heteropolysaccharides, composed of galactose (Gal), glucose (Glu), arabinose (Ara), rhamnose (Rha), and mannose (Man) [148].

The contents of *M. charantia’s* polysaccharides were shown to be influenced by several conditions and classified into two main fractions [149]. One fraction of the *M. charantia* polysaccharide consisted of an acidic, branched heteropolysaccharide (MCBP) mainly composed of Man, galacturonic acid (GalA), Rha, Glu, Gal, xylose (Xyl), and Ara, whereas the rest of the fraction consisted of a pectic polysaccharide (PS) [147,150].

The MCBP fraction possessed antioxidant, *α*-amylase inhibition, and angiotensin-converting enzyme (ACE) inhibition functions [64,150]. Antioxidants remove potentially damaging oxidative agents, which is pivotal to maintaining optimal health, whereas *α*-amylase inhibition delays starch digestion by completely blocking access to the active site of the *α*-amylase enzyme. This action induces weight loss and reduces blood glucose excursions caused by CHO consumption, thus leading to the control and reduction of metabolic comorbidities [151]. This slowed absorption of CHO through the inhibition of enzymes responsible for digestion is one of the many ways in which bitter melon can assist in the control, treatment, and prevention of T2DM and AD [151]. ACE inhibitors aim to lower blood pressure by preventing the production of angiotensin II, a peptide responsible for inducing vasoconstriction of blood vessels. When ACE inhibitors are lacking, the heart contracts against a higher afterload, reducing the stroke volume and causing the release of various hormones known to further increase heart work [152]. Thus, the natural production of ACE inhibitors through the ingestion of biochemically active polysaccharides from bitter melon can potentially achieve cost-effective methods for the treatment and prevention of T2DM and AD [152]. Recently, a water-soluble polysaccharide (MBP) component isolated from *M. charantia*, mainly composed of Ara, Xyl, Gal, and Rha, demonstrated significant hypoglycemic effects, crucial for efficiently controlling T2DM [150]. By doing so, complications often found in uncontrolled T2DM, such as blurred vision, difficulty concentrating, confused thinking, slurred speech, numbness in extremities, and drowsiness are significantly reduced [152]. Complications in relation to AD need further research.

In another study, *M. charantia* polysaccharides were found to improve oxidative stress, hyperlipidemia, inflammation, and apoptosis in myocardial ischemia through the inhibition of the NF-kB signaling pathway [153,154]. *M. charantia* polysaccharides were also found to enhance total volatile fatty acids production, modulate the rumen fermentation pathway, and influence the cellulolytic bacteria population, promoting overall healthy digestion [155]. This is due to the plant’s ability to target both Aß and tau proteins present in the brain. Both findings are relevant to T2DM and AD as these properties hold major importance for each of these metabolic disease states; however, more research on the clinical application of bitter melon is needed.

### 6.2. Proteins and Peptides

Proteins and peptides are other major functional components in both the fruit and seeds of *M. charantia* [64]. Various types have been isolated from different parts of bitter melon, such as polypeptide-p and peroxisomes [85,86,87,88,89,90,91]. These proteins exhibit RNA N-glycosidase activity, PAG activity, DNase-like activity, phospholipase activity, superoxide dismutase activity, anti-tumor, anti-cancer, and immunosuppressive and anti-microbial activity. The hypoglycemic-related activities of the bioactive proteins and peptides of bitter melon are discussed in the next section [85,86,87,88,89,90,91].

#### 6.2.1. Polypeptide-P

One of the main compounds isolated from the medicinal bitter melon plant is polypeptide-p [3]. This peptide is a carbohydrate-binding protein secreted by plant cells, playing a significant role in cell recognition and adhesion reactions, and lowering blood glucose levels [64]. When injected subcutaneously, polypeptide-p acts as an insulin-like hypoglycemic protein [84]. By mimicking the action of human insulin and binding to the INS receptor, plant-based insulin may be used as a replacement in patients with T1DM [92]. In a recent study, the 498 bp gene sequence coding was cloned for the *M. charantia* polypeptide-p and when given to alloxan-induced diabetic mice, lowered blood glucose to controlling methods [93]. Through a better understanding of this polypeptide-p’s actions, this knowledge can be applied to the treatment of T2DM and AD.

#### 6.2.2. Peroxidase

There are several other polypeptides that have been isolated from the fruit, seeds, and tissues of *M. charantia*, including peroxidase [94,95,96]. Peroxidase plays an important protective function in T2DM complications by decreasing oxidative stress and removing toxicity from peroxides and thus converting them into non-toxic substances [97]. Oxidative stress is involved in the pathogenesis of diabetic nephropathy as free radical production exceeds the antioxidative mechanisms to overcome its detrimental effects [97]. These protective functions provide the same benefits toward the pathology of AD, as the pathogenesis of these two metabolic disease states are seemingly related. There are many other important polypeptides within *M. charantia,* including Momordica cyclic peptides, trypsin inhibitors, cystine knot peptides, RNase MC2, antifungal protein, and MCha-Pr. Overall, peroxidase is just one of the many polypeptides within bitter melon that can potentially improve both T2DM and AD [94,95,96,97].

### 6.3. Saponins and Terpenoids

Saponins are a class of glycosides that are responsible for reducing blood lipids, lowering the blood glucose response, and decreasing certain cancer risks [64,98]. They are widely distributed in a variety of plants and are also some of the key active ingredients within several different drugs on the market today [64,99]. All saponins are composed of sugar and aglycone, with the difference lying in the structure of aglycones [64]. In *M. charantia,* saponins can be found within the roots, stems, leaves, and fruit of the plant [64]. Saponins contain a CHO moiety attached to a triterpenoid or steroids, which is another major chemical constituent of *M. charantia,* often referred to as cucurbitanes, which are known for their bitterness in taste and toxicity [64,98]. Cucurbitacins are highly oxygenated, tetracyclic, triterpenic plant substances derived from the cucurbitane skeleton that demonstrate antidiabetic and hypoglycemic bioactive activity [64,100]. Cucurbitane-type compounds, such as goyaglycosides and goyasaponins, have been isolated from the methanolic extract of *M. charantia* fruits, as well as triterpenoids that showed blood-glucose-lowering effects in diabetic mice [64,101]. One study found that these compounds of *M. charantia* have hypoglycemic effects in vivo as well [99,102]. Four of these triterpenoids of *M. charantia* have been identified to possess AMP-activated protein kinase activity, which is a notable hypoglycemic mechanism [3,103]. This evidence highlights the potential of bitter melon in controlling, preventing, and treating T2DM and AD through its bioactive compounds.

#### Charantin

One of the major compounds isolated as a saponin from bitter melon and identified as a hypoglycemic agent includes charantin, a cucurbitane-type triterpenoid [93,104]. In recent research, charantin was a viable option to treat diabetes and showed the potential to replace treatment entirely [105]. Some evidence has shown that the compound has the capability to be more effective than the oral hypoglycemic agent, tolbutamide [17]. In recent a study, two aglycones of charantin were isolated and identified as sitosterol and stigmastadienol glycosides. When tested separately for their hypoglycemic effects in vivo, these two components did not induce any significant changes in blood glucose levels [102,106]. This suggests that charantin may contain other specific factors that have yet to be uncovered that could be identified for the hypoglycemic activity observed in diabetes [3]. Future research is necessary to determine the effect on the pathology of AD.

### 6.4. Flavonoids and Phenolic Compounds

Flavonoids and phenolic compounds are vital components of *M. charantia* that are beneficial to T2DM and AD prevention and treatment [159,160]. Flavonoids can be further classified into six subclasses: flavanols, flavones, flavanones, isoflavones, flavanols, and anthocyanidins [64,132]. They are considered a class of biologically active secondary metabolites of plants responsible for the smell and pigment of flowers and hold several antiviral, anti-allergic, antibacterial, and anti-inflammatory functions [132,161]. Flavonoids can improve the pathogenesis of T2DM, AD, and its complications through the regulation of glucose metabolism, expression of hepatic enzyme activities, and lipid profile [132]. *M. charantia* contains naturally occurring flavonoids with antidiabetic potential including quercetin, rutin, kaempferol, isorhamnetin, and genistein [64,162].

#### 6.4.1. Quercetin

Quercetin is the most abundant flavonoid in human dietary nutrition and acts as the base for the formation of other flavonoid skeletons, such as naringenin, rutin, and hesperidin [107]. With antioxidant, anti-inflammatory, and antiapoptotic effects, quercetin exhibits the potential to treat both T2DM and AD [102,108,109,110]. Quercetin is involved in several biological actions such as glucose homeostasis, insulin-sensitizing and secretion, glucose utilization in peripheral tissues, and the inhibition of intestinal glucose absorption [111]. In skeletal muscle cells, quercetin increases glucose uptake through the stimulation of GLUT4 translocation by activating the AMPK pathway in glucose homeostasis [108]. In hepatocytes, quercetin activates the AMPK pathway by suppressing glucose-6-phosphatase (G6Pase), which in turn lowers hepatic glucose production [108]. A recent study found that quercetin heightened glucose-induced insulin secretion and preserved β-cell function and viability from H_2_O_2_^−^-induced oxidative damage in INS-1 cells [3,109]. These effects were modulated by phosphorylation of extracellular signal-regulated kinase (ERK1/2), suggesting that ERK1/2 activation was involved in quercetin’s mechanism of action [109]. In another study, quercetin improved glucose and lipid metabolism, alleviated hepatic histomorphological injury, and reduced gluconeogenesis in STZ-induced diabetic rats [110]. This was likely associated with the upregulation of SIRT1 activity, as well as its effect on the Akt signaling pathway, showing therapeutic potential for T2DM and obesity [110,111].

Vascular complications are the main cause of morbidity and mortality rates in diabetes patients [112]. In STZ-induced diabetic rats, the administration of quercetin prevented the advancement of diabetes-induced hypertension and negated diabetes-induced vasoconstriction [79]. These outcomes were likely due to the inhibitory effects of quercetin on inflammatory pathways, via NF-κB and by reducing TNF-α and CRP levels in the aorta of diabetic rats [79].

Quercetin may have neuroprotective effects in diabetic peripheral neuropathy [113]. There have been several in vivo and in vitro studies that demonstrated these neuroprotective effects [113,114,115]. One recent study found that high levels of glucose disrupted the proliferation of rat RSC96 cells and primary rat Schwan cells, including suppression of beclin-1 and light chain (LC3), which are the biomarkers for autophagy, and reducing the number of autophagosomes in both cell types [113]. However, after treatment with quercetin, these effects were attenuated [113]. In another study, the supplementation of quercetin reversed cognitive decline in mice fed a high-fat diet [114]. This was associated with altered signaling of Nrf2, which improved overall cognitive function [114]. Furthermore, it was reported that quercetin has the potential to decrease oxidative stress and diminish inflammation and protein glycation in the brain of diabetic rats [115]. These consequences may be related to the upregulation of glyoxalase, which is a ubiquitous cellular enzyme that participates in the detoxification of the cytotoxic byproduct of glycolysis and is believed to have a role in the pathogenesis of diabetic encephalopathy [115].

The favorable effects of quercetin on AD were also established in both cell and animal studies [116,117,118,119,120]. In cultured neurons, pretreatment with quercetin enhanced Aβ1-42-induced protein oxidation, lipid peroxidation, cytotoxicity, and apoptosis. However, high doses had the opposite effect with non-neuroprotective and toxic effects [116]. In one study, quercetin extended the lifespan and supported the climbing ability of AD flies [117]. Cell-cycle-related proteins were disrupted by Aβ accumulation, therefore allowing quercetin to improve cell-cycle-related signaling pathways [117]. In another study utilizing a triple transgenic AD (3xTg-AD) mouse model, a 3-month treatment with quercetin reduced extracellular β-amyloidosis and enhanced microglial and astroglial activation in the brain, as evidenced by diminished levels of Aβ1-40, Aβ1-42, and BACE1-mediated cleavage of APP [118]. In addition, learning and memory were ameliorated [124]. The administration of quercetin to APPsw/ PS1dE9 mice improved learning and memory deficits and reduced plaque levels in comparison to control mice [119]. Quercetin accomplished these protective effects by reducing mitochondrial inhibition through the activation of AMPK [119]. Furthermore, recent research unveiled the potential anti-inflammatory role of quercetin in AD mice [120]. Quercetin treatment diminished β-amyloid plaque accumulation as well as reduced IL-1β/COX-2/iNOS proinflammatory signaling in the hippocampal CA1 region of 3xTg-AD mice [120]. Further research is needed to determine the exact effects of quercetin on both AD and T2DM to best utilize bitter melon in medical preventative practice.

#### 6.4.2. Rutin

Rutin is another flavanol found in *M. charantia*. Its many biological effects include antioxidant, anti-inflammatory, antihyperglycemic, and neuroprotection, all of which support a potential use in the prevention and treatment of T2DM, AD, and associated complications [3,121,122]. For example, in nicotinamide- (NA-) STZ-induced diabetic rats, rutin improved glucose tolerance and reduced serum glucose levels. Rutin also improved the lipid profile, including LDL-cholesterol, VLDL-cholesterol, and triglycerides (TGs) [123]. All these changes were accompanied by an improvement in the oxidative status of diabetic rats.

The possible mechanisms for the antihyperglycemic and antihyperlipidemic effects of rutin were further investigated [3]. In one study, rutin reduced hepatic glucose output through the decreased activity of G6Pase and glycogen phosphorylase, as well as the increased activity of hepatic hexokinase activity [123]. Furthermore, a reduction in glucose levels was accomplished by improving glucose uptake by tissues [122]. Indeed, a recent study showed that rutin decreased blood glucose levels in insulin-resistant mice through the amplification of insulin-dependent receptor kinase (IRK) activity and GLUT4 translocation [123]. In another study, a protective effect of rutin in the livers of db/db mice was revealed through the activation of the IRS2/PI3K/Akt/GSK3β signal pathway, improving hepatocyte proliferation, and decreasing the generation of AGEs, thus making it a useful component towards the treatment and prevention of metabolic complications [124].

Recent evidence has revealed a role of PPARγ expression in adipose tissue and skeletal muscle. Stimulation of PPARγ expression in these tissues enhances insulin sensitivity and increases glucose uptake [123,125]. Treatment with rutin also increased β-cell viability and reduced glucotoxicity through the activation of AMPK and IRS2 signaling [126]. In one study, rutin demonstrated the capacity to improve insulin secretion in isolated rat pancreatic islets [123]. Through this evidence, rutin was found to successfully lower the formation of ROS, AGE precursors, sorbitol, and pro-inflammatory cytokines, thus making it a viable treatment and preventative option for T2DM [108,127].

Other antidiabetic effects of rutin include the reduction of CHO absorption from the small intestine, suppressing gluconeogenesis, activating insulin secretion from β cells, and protecting the islets of Langerhans from degenerative processes [160]. Rutin was also found to significantly minimize oxidative stress through the suppression of inflammatory cytokines in STZ-induced diabetic rats [127]. Therefore, rutin’s ability to enhance glucose uptake by peripheral tissues, improve insulin resistance, suppress gluconeogenesis in the liver, and stimulate insulin secretion make bitter melon a viable remedial option for naturally controlling blood sugar levels [3,123,124,125,126]. Further research on rutin will be beneficial for both medicinal and pharmaceutical purposes in the future.

Rutin has also demonstrated significant therapeutic potential for AD [128]. Through the reduction of inflammatory markers of neurodegeneration, reducing oxidative stress relating to neuronal cell loss, and preventing Aβ aggregation, rutin has the potential to have a significant effect on the prevention and treatment of AD [124]. In a study using APPswe (APP Swedish mutation) cells, rutin prevented Aβ25-35 fibril formation and slowed the activity of BACE [129]. Rutin also improved cell viability, while also lowering GSH levels induced by the overexpression of APP in APPswe cells [129]. In a similar study, rutin inhibited Aβ42 fibrillization and improved Aβ42-induced cytotoxicity in SH-SY5Y cells [32]. Additionally, rutin decreased mitochondrial damage, reduced the formation of ROS, GSSG, NO, iNOS, and proinflammatory cytokines, and promoted the activities of SOD and catalase [124]. In a study using Aβ-injected rats, the administration of rutin significantly improved memory through the activation of the MAPK pathway and brain-derived neurotrophic factor (BDNF) gene expression and declined oxidative stress and neurotoxicity induced by Aβ [130,131].

#### 6.4.3. Kaempferol

Kaempferol is a non-toxic flavonoid with several medicinal effects benefitting both T2DM and AD [133]. Some of the major antidiabetic effects of kaempferol include improving AMP-activated cellular protein expression and activation, reducing cellular apoptosis by suppressing caspase 3 activities, and increasing the production and secretion of insulin from β cells [133,134]. Additionally, kaempferol increases glucose uptake by the cells through protein kinase C and PI3K pathways and enhances the synthesis of glucose transporter proteins [135]. Furthermore, kaempferol significantly reduces serum HbA1c levels and fasting blood glucose while enhancing insulin sensitivity when administered orally [132,135]. Kaempferol also has been shown to lessen the expression of PPARγ mediated through regulating AMPK activation [136]. Moreover, kaempferol improved the diabetic state of STZ-induced mice through glucose metabolism in skeletal muscle and suppression of hepatic gluconeogenesis [137]. In another study, researchers found that kaempferol reduced diabetic nephropathy in NRK-52E and RPTEC cells by decreasing RhoA/Rho-kinase mediated pro-inflammatory signaling (i.e., TNF-α, IL-1β, and TGF-β1) [137]. Taken together, the results of these studies highlight the potential of kaempferol as a therapeutic agent for the treatment of diabetes. However, further research is needed to gain a better understanding of the role of kaempferol in AD.

#### 6.4.4. Isorhamnetin

Isorhamnetin is another bioactive compound within *M. charantia* that has anti-obesity and antidiabetic effects that may also be applied to AD prevention and treatment [138]. In a recent study, isorhamnetin was administered orally for 10 days to STZ-diabetic mice at a dose of 10 mg/kg or 20 mg/kg and successfully demonstrated a reduction in oxidative stress and hyperglycemia [139]. In another study, isorhamnetin was not only able to reduce blood glucose levels, but also decreased the aggregation of sorbitol levels on the lens of the oculus, the sciatic nerve, and red blood cells, which are all common complications of uncontrolled T2DM in humans. [140]. Any bioactive effect that prevents the complications of either T2DM or AD is beneficial for the overall treatment and in the understanding of these diseases. Further research is needed on isorhamnetin to best identify the precise effects of its role in bitter melon.

### 6.5. Other Components

There are several other components within bitter melon that have been identified and isolated from the plant including unsaturated fatty acids (FA), alkaloids, amino acids (AA), vitamins, and minerals [3,163,164,165]. There is a relatively high amount of unsaturated FA components with monounsaturated fatty acids (MUFAs) making up about 20.1% of total fatty acid content and polyunsaturated fatty acids (PUFAs) making up roughly 64.3% [64]. Altogether, nine different types of unsaturated FA have been identified in bitter melon [166]. These unsaturated FAs are known to enhance insulin sensitivity, thereby improving metabolic disease states such as T2DM and AD [166]. Through acid hydrolysis and AA analysis, the total content of AA is estimated at 11.99% and 2.36% for free AA [167]. AA are important bioactive components of bitter melon as they provide nutrients to improve blood pressure, hyperglycemia, visceral obesity, and abnormal cholesterol or TG levels. Additionally, *M. charantia* is a considerable source of vitamins and minerals, including ascorbic acid [168,169,170]. Ascorbic acid appears to lower blood glucose increase insulin synthesis and secretion, and enhance insulin sensitivity, all of which are beneficial in T2DM, AD, and other metabolic diseases [73,170]. The effects of bioactive compounds in bitter melon on AD and T2DM are given in Table 1. To fully understand the impact of bitter melon on T2DM and AD, further research will be necessary to uncover the vast effects of these other components within the plant.

## 7. Incorporating Bitter Melon into the Diet

Bitter melon can be easily incorporated into one’s diet to maintain control of blood glucose levels. The bioactive components within *M. charantia* allow for these hypoglycemic effects to occur. Information regarding the proper ways to consume this nutritious plant is necessary to achieve the safest and best results.

### 7.1. Defining Medical Nutrition Therapy

Clinical nutrition is focused on the prevention, diagnosis, and management of nutritional changes in patients with chronic diseases and conditions. As research reveals the importance of a well-balanced diet filled with a variety of vitamins and minerals, it has become apparent that nutrition plays a much bigger part in overall health. In relation to chronic disease and illness, medical nutrition therapy has become the forefront of treatment, making nutrition critical for successful patient care and recovery. Medical nutrition therapy utilizes the different bioactive components within foods to best treat patients in the most cost-effective and safe way possible. The importance of this therapy is further exemplified by its gravitation towards its individualized care in determining which foods best manage and treat ailments of certain chronic diseases or metabolic disorders.

### 7.2. Impact of Bitter Melon as Medical Nutrition Therapy

As elements within plants such as *M. charantia* are unraveled, and their medicinal effectiveness better understood, the more we can utilize their effects in actual practice. As previously discussed, the medicinal benefits of bitter melon extend beyond the fruit itself, but to its stem, leaves, and roots as well [3]. Therefore, the entire plant can be used to help manage the complications relating to T2DM, as well as prevent or slow the progression of the signs and symptoms contributing to the pathogenesis of AD [3,55]. The phytochemical components within bitter melon act as insulin to help reduce blood sugar levels and demonstrate certain neuroprotective effects through its bioactive additives including terpenoids, glycosides, flavonoids, phenolic, and charantin. Furthermore, as AD and T2DM share common etiologies, the consumption of bitter melon carries significant value as a treatment and control option for both diseases.

### 7.3. Incorporating Bitter Melon into the Diet

*M. charantia’s* ability to lower blood glucose levels is a significant representation of the power of food and its impact on the population’s overall health. The fruit itself can either be consumed raw or cooked, however many prefer it cooked to tone down the bitter flavor. One should consult with a primary care provider before incorporating bitter melon into one’s diet, as consuming this fruit may cause very low blood sugar levels when combined with traditional diabetes medicine. Bitter melon is not safe for children or for pregnant or breastfeeding women to consume. Further research is needed regarding any possible interference with the bioavailability of other nutrients as well as its effect on the mechanisms of various medications one may be prescribed. Nevertheless, bitter melon is a safe, effective, and affordable option to naturally control blood glucose levels. As a result, the moderate consumption of this nutrient-rich food can help in the control of T2DM and possibly delay the progression of AD.

## 8. Summary

Overall, T2DM and AD are complex metabolic disorders occurring at alarmingly high rates with substantial social and economic burdens [19]. The lack of successful therapeutic treatment options in the management of AD and long-term diabetes requires the development of safe and effective complementary approaches, such as the use of the *M. charantia* plant [19]. The various bioactive compounds within bitter melon have piqued the interest of researchers and have led to the exploration of the therapeutic potential of this vine fruit [19]. Recognizing the molecular mechanisms of action underlying the antidiabetic and neuroprotective effects of bioactive compounds in cell cultures and animal models of T2DM and AD is the first step in uncovering *M. charantia’s* potential action on these metabolic disorders [19].

**Figure 4:**
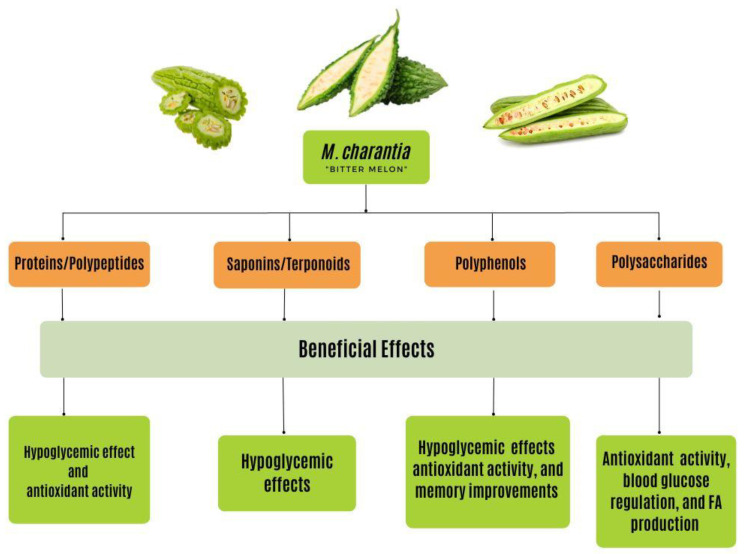
The beneficial effects of bioactive compounds found in M. charantia have various effects on chronic disease states, including T2DM and AD. These effects include hypoglycemic, antioxidant, blood glucose regulating, FA production, and memory-enhancing effects. These effects stem from the proteins, saponins, polyphenols, and polysaccharides found within this plant. Through the consumption of this plant, the beneficial effects are activated from the bioactive compounds. T2DM, type 2 diabetes mellitus; AD, Alzheimer’s disease.

Published data emphasize the prospective beneficial effects of bioactive compounds (Figure 4) on lowering hyperglycemia, magnifying insulin secretion, amplifying β-cell function, reducing Aβ accumulation, and strengthening cognitive function [19]. In this paper, the role of the major bioactive compounds of bitter melon was explored, including polysaccharides; proteins and peptides such as polypeptide-p, and peroxidase; saponins and terpenoids such as charantin; and flavonoids and phenolic compounds such as quercetin, rutin, kaempferol, and isorhamnetin to fully grasp its significance in the treatment and prevention of both T2DM and AD [3,64,92,157]. As research on the bioactivities of *M. charantia* continues to develop, a better understanding of the antioxidant, anti-inflammatory, and antiapoptotic properties and their mechanisms is necessary for the advancement of plant-based alternative medicine, the curation of new and effective drugs, and the growth of medical nutrition therapy [19,64]. Thus, clinical studies of the bioactive components should be the focus of future research [64]. In doing so, the relationship between the structure and mechanisms of the various functional components within bitter melon will be further clarified [64].

Most research conducted on the bioactive compounds of *M. charantia* has produced controversial results, which may be due to several factors including experimental design, dosage, and types of bioactive compounds examined [19]. Additionally, the possible complications from long-term consumption on the human body have not been extensively explored [64]. Therefore, carefully designed clinical trials will be needed to produce relevant evidence for the potential therapeutic utilization of bioactive compounds within *M. charantia* in the treatment of T2DM and AD [19].

## 9. Conclusions

Recognizing the utilization of food as medicine is an important concept in nutrition sciences as reputable research continues to grow within this field [3]. Throughout many centuries, *M. charantia* has been used as a method of alternative medicine and dietary supplement for treating symptoms and conditions related to what we know today as diabetes [3]. Bitter melon is characterized as a multipurpose plant worthy of treating several diseases known to mankind and has been substantially studied across the globe for its powerful medicinal properties [2,3]. This may be due to the plant’s various medicinal elements that act either separately or congruently to exert their medicinal effects [3,170]. In relation to DM, the hypoglycemic properties of bitter melon are what bring this plant its justified attention [3]. In relation to AD, the various antioxidant properties are what provide protection to both cognitive function and cholesterol levels within the brain. These different bioactive compounds seem to exert their beneficial effects through several mechanisms of action to best control and treat DM and AD [3]. 

Although this review includes an elaborate discussion of biochemical and animal studies on *M. charantia*, these studies are flawed by small sample size, lack of control, and poor study designs. This paper advocates the need for certain improvements to be made in study design for clinical trials in relation to adequate sample size and statistical power. By doing so, the safety and efficacy of *M. charantia* as a natural nutritional treatment for DM and AD can be suggested with scientific evidence [3]. Furthermore, *M. charantia* may have a greater effect on ethnic minorities who have a higher incidence of diabetes but prefer natural treatment based on cultural beliefs [3].

In conclusion, the application of bitter melon in medicine remains in the initial processing stages, as scientists continue to uncover its numerous health benefits from its bioactive constituents [64]. *M. charantia* not only has the potential to be a safe and effective therapeutic method for individuals suffering from the complications of T2DM and AD but also as a cost-effective option to ease the economic and social burden these metabolic disorders have on the populations worldwide. Further research is needed to determine the exact effects of bitter melon on humans from a clinical standpoint.

## Figures and Tables

**Table 1:** Classification of *M. charantia*.

Kingdom:	Plantae
Common Name:	Bitter melon, bitter gourd, karela.
Order:	Cucurbitales
Species:	*M. charantia*
Genus:	*Momordica*
Family:	Cucurbitaceous
Class:	Magnoliopsida
Division:	Magnoliophyta

The classification of the medicinal plant, *M. charantia*, otherwise known as bitter melon. Through further understanding of the plant’s origin, one may assess its effects more effectively.

**Table 2:** Effects of bioactive compounds in bitter melon on AD and T2DM.

Major Bioactive Compounds	Bioactive Functions	Mechanism of Action	Beneficial Effects	Distribution	Reference
Proteins/Peptides: (1) Polypeptide-P (2) Peroxidase	RNA N-glycosidase, polynucleotide adenosine glycosidase (PAG), DNase-like, phospholipase, superoxide dismutase, anti-tumor, immune suppression, antimicrobial	(1) Binds to INS receptor (2) ↓ oxidative stress; ↓ toxicity	(1) Hypoglycemic effect (2) Neutralization by the antioxidant activity	Seed	[3,64,84,85,86,87,88,89,90,91,92,93,94,95,96,97]
Saponins/Terpenoids: Charantin	Antioxidant, antidiabetic, hypoglycemic, anticancer, hypolipidemic, antiviral	AMP-activated protein kinase activity; ↓ blood lipid levels; ↓ blood glucose response	Hypoglycemic effects	Fruit, root, seed, stem, and leaves	[3,17,64,93,98,99,100,101,102,103,104,105,106]
Polyphenols: (1) Quercetin	Antioxidant, anti-inflammatory, antiapoptotic, and immune-enhancing	(1) ↓ Lipid peroxidation & ↓ Oxidative stress via ERK1/2 activation; ↑ activation of AMPK; ↓ activity of G6pase; ↓ TNF-*α*, CRP, NF- κB; ↓ Aß1-40, Aß1-42, and BACE1	(1) ↓ Aß-42-induced apoptotic cell death and cell toxicity; ↑ GLUT4 translocation; ↓ hepatic glucose production; ↓ diabetes-induced HTN and vasoconstriction; ↑ Learning and memory function	Fruit, pericarp, and seed	[3,79,102,107,108,109,110,111,112,113,114,115,116,117,118,119,120]
(2) Rutin		(2) Free-radical scavenger activity; via PI3K, atypical protein kinase C and MAPK pathways; ↑ IRK activity, ↑ GLUT4 translocation; ↓ activation of MAPK pathway, ↑ BDNF gene expression	(2) ↓ formation of Aß fibrils and disaggregated Aß fibrils, ↓ neurotoxicity; ↑ glucose; ↓ bg; ↓ Aß-induced learning and memory deficits, ↓ Aß-induced neurotoxicity		[3,32,108,121,122,123,124,125,126,127,128,129,130,131]
(3) Kaempferol		(3) ↑ production and secretion of insulin from ß cells; ↑ glucose uptake via protein kinase C and PI3K pathway; regulation of AMPK activation; ↓ of hepatic gluconeogenesis; ↓ RhoA/Rho kinase-mediated pro-inflammatory signaling	(3) ↓ cellular apoptosis; enhance synthesis of glucose transporter proteins; ↓ HbA1c and fasting blood glucose; ↑ glucose metabolism; ↓ diabetic neuropathy		[132,133,134,135,136,137]
(4) Isorhamnetin		(4) Activation of JAK2/STAT pathway and promotion of GLUT4 translocation; Activation of NO/GC/cGMP pathway and cyclooxygenase pathway	(4) Hypoglycemic effect; ↓ oxidative stress, ↓ blood glucose levels; ↓ sorbitol aggregation		[138,139,140]
Polysaccharides	Antioxidant, antidiabetic, immune enhancing, neuroprotective, antitumor, antimicrobial, hypoglycemic, and anti-inflammatory	Inhibition of *α*-amylase, ACE, and NF-κB signaling pathway	↓ oxidative stress; regulates blood glucose; ↑ volatile FA production	Various parts of the plant	[64,141,142,143,144,145,146,147,148,149,150,151,152,153,154,155]

The active bioactive compounds within bitter melon have significant beneficial effects on both AD and T2DM through their various mechanisms. These components are distributed throughout the *M. charantia* plant, with some parts producing different effects than others. ACE, angiotensin-converting enzyme inhibitors; AMPK, activated protein kinase; BACE1, beta-site amyloid precursor protein cleaving enzyme 1; BDNF, brain-derived neurotrophic factor; CRP, C-reactive protein; ERK1/2, extracellular signal-regulated kinase; FA, fatty acids; HbA1C, hemoglobin A1C; GLUT4, glucose transporter type 4; G6Pase, Glucose-6-phosphatase; HTN, hypertension; JAK2/STAT, Janus kinase/signal transducers and activators of transcription; MAPK pathway, mitogen-activated protein kinase; NF-*κ*B signaling pathway, nuclear factor kappa B; PI3K pathway, phosphatidylinositol-3 kinase; TNF, tumor necrosis factor; ↑: increase; ↓: decrease

## Data Availability

Not applicable.

## Abbreviations

AD, Alzheimer’s disease; ACE, angiotensin-converting enzyme; AGE, advanced glycation end products; AMPK, activated protein kinase; AKT, protein kinase B; Ara, arabinose; Aβ peptide, amyloid plaques; APP, amyloid precursor protein; APPswe, APP Swedish mutation cells; BACE-1, β-site APP cleaving enzymes 1; BACE, ß-secretase; BBB, blood brain barrier; BDNF, brain-derived neurotrophic factor; Ca, calcium; CaM- CaMKIV, calcium/calmodulin-dependent protein kinase IV; CRP, C-reactive protein; COX-2, cyclooxygenase-2; *M. charantia*, Momordica charantia; MCL, *M. charantia* lectin; CNS, Central Nervous System; DM, diabetes mellitus; ER, endoplasmic reticulum; ERK1/2, extracellular signal-regulated kinase; Fe, iron; Gal, galactose; GalA, galacturonic acid; Glu, glucose; GLUT4, glucose transporter type 4; GSK3ß, glycogen synthase kinase 3ß; G6Pase, glucose-6-phosphatase; HbA1c, glycated hemoglobin; HDL, high-density lipoprotein; IDE, insulin-degrading enzyme; IL-6, interleukin-6; IL-1β, interleukin-1β; iNOS, nitric oxide synthase; IR, Insulin receptors; IRK, insulin-dependent receptor kinase; IRS, insulin receptor substrate; JNK, c-Jun N-terminal kinase pathway; K, potassium; LC3, light chain; LDL, low-density lipoprotein; Man, mannose; Mg, magnesium; MBP, water-soluble polysaccharide; MCBP, branched heteropolysaccharide; MCI, mild cognitive impairment; MUFA, monounsaturated fatty acids; NA, nicotinamide; NFT, neurofibrillary tangles; NF-κB, nuclear factor-kappa B; NGF, nerve growth factor; Nrf2, nuclear factor erythroid 2-related factor 2; OCD, obsessive-compulsive disorder; P, phosphorus; PAG, periaqueductal gray; PEPCK, phosphoenolpyruvate carboxykinase; PI3-kinase, phosphatidylinositol-3 kinase; PUFA, polyunsaturated fatty acids; PPAR, peroxisome proliferator-activated receptors; PS, pectic polysaccharide; p38-MAPK, p38-mitogen-activated protein kinase; RAGE, the receptor for advanced glycation end products; RCT, randomized control trial; Rha, rhamnose; RIP, ribosome-inactivating proteins; RNS, reactive nitrogen species; ROS, reactive oxygen species; SARK, stress-activated protein kinase; SIRT1, silent mating type information regulation 2 homolog; SOD, superoxide dismutase; STZ, streptozotocin; T1DM, type 1 diabetes mellitus; T2DM, type 2 diabetes mellitus; TG, triglyceride; TNF-α, tumor necrosis factor-α; US, United States; VLDL, very low-density lipoprotein; Xyl, xylose; Zn, zinc; α-MMC, alpha-momorcharin; β-MMC, momorcharin; 3xTg-AD, triple transgenic AD mouse model.
